# Rescuing Auditory Temporal Processing with a Novel Augmented Acoustic Environment in an Animal Model of Congenital Hearing Loss

**DOI:** 10.1523/ENEURO.0231-21.2021

**Published:** 2021-07-14

**Authors:** Adam C. Dziorny, Anne E. Luebke, Luisa L. Scott, Joseph P. Walton

**Affiliations:** 1 Department of Biomedical Engineering, University of Rochester School of Medicine, Rochester, NY 14642; 2 Department of Pediatrics, University of Rochester School of Medicine, Rochester, NY 14642; 3 Department of Neurobiology and Anatomy, University of Rochester School of Medicine, Rochester, NY 14642; 4 Cognosetta, Inc, Tampa, FL 33620; 5 Departments of Communication Sciences and Disorders, Medical Engineering and Global Center of Speech and Hearing Research, University of South Florida, Tampa, FL 33620

**Keywords:** augmented acoustic environment, hearing loss, inferior colliculus, mouse, temporal processing

## Abstract

Congenital sensorineural hearing loss (SNHL) affects thousands of infants each year and results in significant delays in speech and language development. Previous studies have shown that early exposure to a simple augmented acoustic environment (AAE) can limit the effects of progressive SNHL on hearing sensitivity. However, SNHL is also accompanied by hearing loss that is not assessed on standard audiological examinations, such as reduced temporal processing acuity. To assess whether sound therapy may improve these deficits, a mouse model of congenital SNHL was exposed to simple or temporally complex AAE. The DBA/2J mouse strain develops rapid, base to apex, progressive SNHL beginning at birth and is functionally deaf by six months of age. Hearing sensitivity and auditory brainstem function was measured using otoacoustic emissions, auditory brainstem response (ABR) and extracellular recording from the inferior colliculus (IC) in mice following exposure to 30 d of continuous AAE. Peripheral function and sound sensitivity in auditory midbrain neurons improved following exposure to both types of AAE. However, exposure to a novel, temporally complex AAE more strongly improved a measure of temporal processing acuity, neural gap-in-noise detection in the auditory midbrain. These experiments suggest that targeted sound therapy may be harnessed to improve hearing outcomes for children suffering from congenital SNHL.

## Significance Statement

Congenital sensorineural hearing loss is common and often results in delays in grammar comprehension, vocabulary retention and speech development secondary to auditory temporal processing deficits. Exposure to an augmented acoustic environment early in life mitigates outer hair cell death and peripheral auditory dysfunction, however no studies have examined early complex acoustic stimuli on neural coding in the central auditory system. In this study we characterize the impact of early hearing loss on auditory temporal processing and compare two types of augmented acoustic exposure on subsequent neural correlates of temporal processing. We show that temporally complex acoustic stimuli are better able to rescue impaired temporal encoding, suggesting that temporally structured acoustic exposure improves neural processing deficits caused by loss of peripheral input.

## Introduction

Early childhood sensorineural hearing loss (SNHL) is a common neurosensory disability causing significant medical, social and financial hardship. The prevalence of moderate-to-profound SNHL in children (>40 dB) is roughly three in 1000, with up to 10% have hearing loss that is considered “profound” (Davidson et al., 1989; Dalzell et al., 2000; Elden and Potsic, 2002; Picton et al., 2005). There are numerous causes of congenital or acquired SNHL, including genetic factors, infectious diseases, or environmental toxins. Beyond hearing threshold deficits seen in children with SNHL, studies have also shown functional deficits in the development of speech and language processing (Needleman and Crandell, 1995; Yoshinaga-Itano et al., 1998; Yoshinaga-Itano, 2003a,b; Wake et al., 2004). Impairments in speech perception, which may give rise to these functional deficits, have been associated with restricted encoding of auditory temporal cues (Snell, 1997).

Psychoacousticians have used the gap detection paradigm to evaluate temporal processing acuity of sounds for >30 years. Gap detection acuity may underlie perceptual boundaries in natural language, such as voiced versus voiceless speech sounds. Minimal gap thresholds (MGT) appear to determine the perceptual boundary in the continuum of voice onset times (VOTs), the intervals between consonant release and the start of vocal cord vibration in consonant-vowel transitions (Elangovan and Stuart, 2008). Gap detection acuity is also linked to speech recognition abilities (Snell et al., 2002), as well as normal language development (Trehub and Henderson, 1996; Benasich and Tallal, 2002). In animal models, gap detection can be assessed using several different behavioral techniques (Giraudi et al., 1980; Hamann et al., 2004), and can also be measured neurophysiologically to assess neural sound encoding (Eggermont, 1995; Walton et al., 1997). Interestingly, nearly all mammals have similar behavioral MGTs, which are on the order of 2–3 ms; the lowest neural MGTs approximate these behaviorally-assessed MGTs (Barsz et al., 2002).

There are several mouse models of congenital SNHL that mimic the different types and progression of hearing loss that occur in humans. The DBA strain, the oldest inbred mouse strain (Beck et al., 2000), contains a mutation in the gene, *Cdh23* (Noben-Trauth et al., 2003), as well as a nucleotide substitution in *Fscn2* that is the cause of the *ahl8* quantitative trait locus (Johnson et al., 2008; Shin et al., 2010). This strain shows a rapid, progressive loss of peripheral function beginning at the onset of hearing (Ralls, 1967; Erway et al., 1993), displaying many of the audiometric characteristics found in infants with progressive SNHL (Willott et al., 1984). DBA mice have early and rapid loss of outer hair cell (OHC) function in a base to apex progression, as measured by distortion product otoacoustic emission (DPOAE) thresholds (Parham et al., 1997).

Previous studies have shown that when newborn DBA mice are exposed to broadband sounds daily during 12-h on/off cycles, improvements are seen in peripheral function (Turner and Willott, 1998; Willott and Turner, 1999), preserving hearing sensitivity and limiting hair cell loss (Willott et al., 2005). The mechanism through which the slowing of the degenerative processes occurs is unknown but perhaps the augmented acoustic environment (AAE) maintains afferent neuronal input to the central auditory system (CAS; Willott et al., 2010). AAE exposure also limits neuron loss in the cochlear nucleus (Willott et al., 2005) and expands the frequency range to which inferior colliculus (IC) neurons are sensitive across the dorso-ventral axis compared with non-exposed mice (Willott and Turner, 2000). When normal-hearing, young adult CBA mice are exposed to AAE no effects, positive or negative, are observed (Willott et al., 2000). Clearly, in mouse models of congenital SNHL, AAE exposure shows promise in ameliorating the effects of rapid, progressive SNHL on loss of hearing sensitivity. However, whether AAE exposure can ameliorate other aspects of auditory dysfunction associated with SNHL has yet to be studied. The goal of the current study was to test the hypothesis that exposure to targeted AAE featuring temporally complex sound features would improve neural correlates of gap encoding in the CAS.

## Materials and Methods

### Animal model

The DBA/2J mouse strain has served as a mouse model of early-onset severe hearing loss for over 4 decades (Ralls, 1967; Willott et al., 1982; Erway et al., 1993). Founder breeding pairs were obtained from The Jackson Laboratory, and our colony was maintained in microisolator facilities in the institutional vivarium. Mice were housed in rodent microisolator cages and provided *ad libitum* food and water. Lights in the room were on a 12/12 h light/dark cycle. Cages were changed at least weekly and mice were monitored for signs of distress by trained vivarium technicians. Breeder pairs were kept separate from experimental mice; only nulliparous mice were used for experiments. Both control and exposed pups were weaned into gender-separated cages between ages postnatal day (P)21 and P28. All mice in this study were between first and fourth generation Jackson Labs breeder mice offspring. All procedures were approved by the University of Rochester’s Committee on Animal Resources and in strict accordance with the National Institutes of Health *Guide for the Care and Use of Animals*.

### AAE exposure

**Figure 1.**
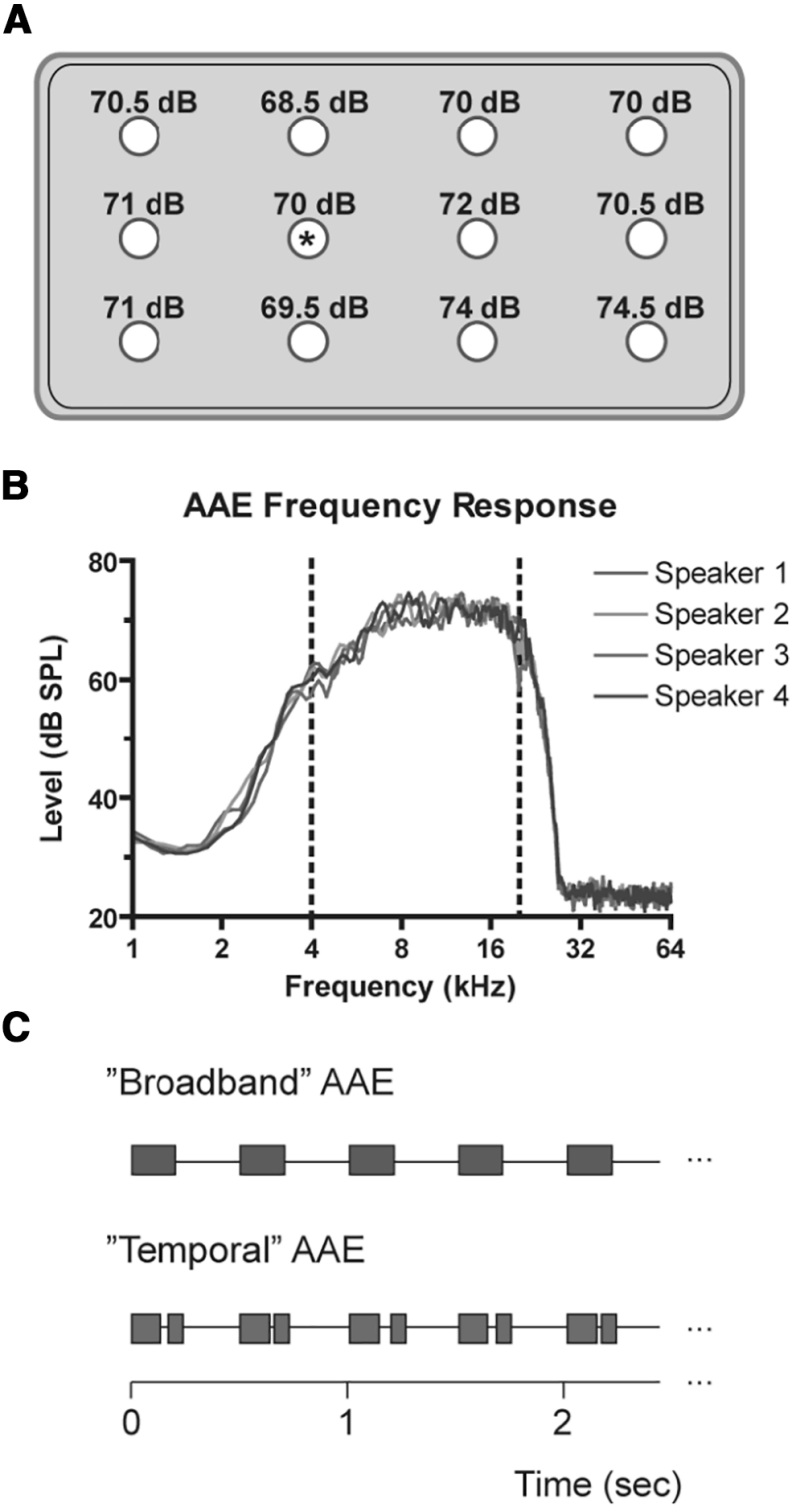
Exposure calibration, stimulus spectrum and temporal pattern. ***A***, Sound levels recorded at different points in the cage in response to calibrated AAE stimulus. Circles denote points drilled into the bottom of a sample cage spaced ∼3.5” apart, through which a calibration microphone was inserted to the approximate height of a mouse off the cage bedding. Asterisk denotes hole calibrated to 70 dB SPL. ***B***, The frequency response spectrum of AAE stimulus, presented through each speaker used in exposure (only one speaker was in use at any given time), demonstrates a flat region (±6 dB) from 4 to 20 kHz (indicated with dashed lines). The spectrum was recorded with a ½” ACO Pacific microphone on a Quest 1900 sound level meter output to an HP/Agilent 35665A spectrum analyzer. Each waveform consisted of 30 averages. ***C***, Schematic of regular AAE and T-AAE exposure. Each exposure was presented twice per second, 200 ms per burst, for 12 h/d. T-AAE stimulus had a silent gap (either 0, 1, 2, 4, 8, or 16 ms in duration) inserted after the first 100 ms.

Mice were exposed using the same amplifier and sound source as described previously (Turner and Willott, 1998; generously provided by Jeremy Turner). Cages were housed inside a sound-attenuating chamber (approximately three feet wide × two feet deep × five feet high) covered with anechoic foam, and the booth itself was housed in a two-way vivarium room. Stimulus presentation via a single Radio Shack Supertweeter was calibrated *in situ* to 70 dB SPL using a Quest 1900 sound level meter and an ACO Pacific ¼” free-field microphone (Fig. 1*A*). The spectrum of regular AAE (R-AAE) exposure was recorded using an HP/Agilent 35665A Spectrum Analyzer (Hewlett Packard). The analysis revealed a wide-band noise ±6 dB from 4 to 20 kHz (Fig. 1*B*). Ambient noise levels were between 39 and 45 dB SPL in this frequency range. Our novel temporal AAE (T-AAE) stimulus was generated from a subsection of the wave file containing our original stimulus (Fig. 1*C*), with additional silent gaps inserted within the noise bursts, as follows. Random gap durations of 0, 1, 2, 4, 8, or 16 ms were inserted into the wave vector 100 ms into each 200-ms noise burst, and the remaining noise burst was shortened by the same gap duration to preserve the 40% duty cycle. The rise/fall times for the AAE gap stimuli were identical to the gap-in-noise stimuli used to assess neural processing. The resulting wave vector was saved and used for T-AAE stimulus presentation (Fig. 1*C*). The spectrum of T-AAE also measured *in situ* was found to be identical to the regular AAE spectrum.

AAE exposure began just after birth, before the onset of hearing at around P10 (Shnerson and Pujol, 1983) matching previous studies in the DBA model (Turner and Willott, 1998; Willott et al., 2000, 2005). Mice were continuously exposed through testing at 30 d after birth (P30), with both exposure groups having the same duration of exposure. This time point was chosen because control mice of this strain already show significant hearing loss by this time (Ralls, 1967; Erway et al., 1993; Turner and Willott, 1998). Control animals were housed in a two-way vivarium room, physically separated from the sound-attenuating chamber described above, while sensory and social exposures were identical to exposed animals including identical cage type, number of cage mates, and similar visual and olfactory stimuli. Other than exposure to acoustic environment, no other exposure difference existed between the control and AAE groups.

### Peripheral auditory assessment

Before all auditory assessments, mice were anesthetized with tribromoethanol. Auditory brainstem responses (ABRs) were recorded using BioSigRP software [version 4.4.1; Tucker-Davis Technologies (TDT)] interfacing with TDT System III hardware. ABR waveforms were recorded in response to tone bursts of 5-ms duration, shaped by a Blackman window, with a 1-ms onset and offset ramp and a cosine gating function. The frequencies tested were 3, 6, 12, 16, 20, 24, 32, and 36 kHz for each animal. Stimuli were presented at a rate of 25/s, with 150 averages per waveform, with replication. Artifact rejection was enabled with a threshold of 7 μV. Each frequency was presented beginning at a level of 80 dB SPL down to 20 dB below threshold, in 5-dB increments. The recorded waveforms were amplified (10,000×), filtered (0.3–10 kHz), and digitized. No mice (control or experimental) responded to test frequencies >24 kHz, and therefore these frequencies were omitted from the ABR analysis.

DPOAEs amplitudes were measured using custom MATLAB (MathWorks) software interfacing with TDT System III hardware, calibrated similar to the ABR acquisition hardware. The speaker transduction tube/ER10B microphone apparatus was lowered into the ear canal of the anesthetized mouse using a micromanipulator under microscopic examination. Two separate placements and recordings were completed on each mouse; if the results differed, a third placement and recording was completed. The pair of matching results was averaged during analysis. DPOAE amplitudes were recorded in response to two simultaneous pure-tone bursts (f_1_ and f_2_) at different frequencies, related with the following ratio: f_2_/f_1_ = 1.25. The lower-frequency tone (f_1_) was presented at 65 dB SPL and the higher-frequency tone (f_2_) was presented at 50 dB SPL. The geometric mean presentation frequencies were from 5.6 to 20.5 kHz. Amplitudes were transformed to the frequency domain and the cubic distortion product (2f_1_ – f_2_) and surrounding noise values were measured. DPOAE responses could not be distinguished from the noise floor above 22 kHz in any group, and these frequencies were not analyzed.

### Auditory midbrain neurophysiology

We recorded neuronal activity in the IC using a 16-channel vertically-oriented electrode (a1x16-3 mm-100-177μm^2^, NeuroNexus Technologies) with 100-μm spacing between pads and impedances of 1–3 MΩ. On the day before recording, we created a surgical craniotomy on anesthetized mice using techniques described previously (Walton et al., 1998). On the day of recording, mice were again anesthetized with tribromoethanol for the duration of the recording session. Electrodes were positioned over the craniotomy and advanced ventrally by a micromanipulator. The electrical output was amplified, filtered and digitized at 25 kHz in a 1.25-ms time window. Neural activity was automatically determined using a 3:1 signal-to-noise ratio (SNR).

Stimuli were generated using DSP software (OpenEX and RPVDS, TDT) and presented through TDT System III hardware to an electrostatic speaker (TDT ES1). The speaker was located at a 60° azimuth contralateral to the recording site. Stimulus presentation was controlled by custom MATLAB routines interfacing through OpenEx interfaced to an RX6. First, search stimuli were presented to locate responsive units and identify events. These stimuli were band-limited noise bursts (3–50 kHz) presented at 70 dB SPL at a rate of 2/s. Second, tone burst stimuli (25 ms in duration, 10/s) were presented to measure frequency response areas (FRAs). The range of frequencies used in this study was 2–64 kHz (500-Hz increments) and the range of levels was 0–85 dB SPL (5-dB increments). Each frequency-level pair was presented five times, with the entire set randomized before presentation. Third, to assess gap-in-noise encoding, noise bursts (3–50 kHz) of 150 ms were delivered at a rate of 2/s. The level was fixed at the start of each run to 80, 70, or 60 dB SPL, as these intensities were predicted to be >20 dB higher than the noise threshold for individual units. Silent gaps were inserted (with 0.25-ms rise-fall) into the noise burst after the first 100 ms of the noise burst (resulting in a gap-in-noise burset). The gap duration was randomly chosen to be one of the following intervals: 0, 1, 2, 4, 8, 16, 32, 64, or 96 ms. Additionally, continuous background noise (CBN) was used to further test the benefits of AAE exposure. CBN (3–50 kHz) was applied to a gap series at a fixed level (+6 dB SNR) whereby the silent gap would also be filled with this CBN (at a level of 6 dB below the gap-in-noise carrier). For each background condition (quiet or CBN) and gap-in-noise stimuli level, each gap duration was repeated 50 times for a total of 500 repetitions (10 gap durations × 50 repetitions per duration).

Spike waveforms were processed in MATLAB using the TDT OpenDeveloper ActiveX controls and passed to AutoClass C v3.3.4, an unsupervised Bayesian classification system that seeks a maximum posterior probability classification, developed at the NASA Ames Research Center. AutoClass scans the dataset of voltage–time waveforms according to custom-specified spike parameters to produce the best fit classifications of the data, which may include distinct single-unit and multi-unit events, as well as noise. To discriminate the signal from noise in the present study, the variance of the background noise was estimated as the quartile range of the first five digitization points of the spike waveform, at these are recorded before the threshold-crossing event. To avoid overloading AutoClass with excessive noise, which leads to overclassification, this noise measure is used to screen the event waveform data, such that only voltage points with absolute values greater than this noise floor were presented for use in the classification. Once the classes had been determined in each channel of data, the events were visualized within a custom MATLAB program and assigned to multi-unit, single-unit, or noise classes. Events were categorized as noise were subsequently discarded, and units with distinct biphasic waveforms and good SNRs were classified as single-units. As most channels recorded information elicited from the spiking of two or more neurons, all recorded units in this paper were considered to be multi-unit activity. Nonetheless, there was no observation of any consistent differences in the receptive fields between single units and multi-unit clusters.

Data analysis was performed as previously described (Leong et al., 2011). FRAs were displayed in a custom MATLAB GUI and analyzed with a multi-step procedure using custom software. FRAs were then used to determine the best frequency (BF), the frequency with the lowest intensity of driven activity, and tuning sharpness. For units with sound driven activity, assessed within the FRA, neural responses to gap-in-noise stimuli were visualized using a custom MATLAB GUI, and minimum gap thresholds (MGTs) were determined using previously published methods (Walton et al., 1998, 2008). Only gap-responsive units (with MGT ≤ 96 ms) for each stimulus condition were included in subsequent analysis of gap detection. Additionally, the analysis focuses on phasic units because tonic units recorded in CBN demonstrated postexcitatory suppression. Because of this postexcitatory suppression, the quiet window responses of tonic units were not strictly a result of the embedded silent gap, making MGT determination highly variable and unreliable.

### Hair cell counts

A subset of cochleae were harvested from mice to perform hair cell counts following 60 d of AAE treatment. Mice were deeply anesthetized with isofluorane, decapitated, and the cochleae were dissected and placed in postfix (4% paraformaldehyde). They were decalcified in 5% EDTA for 72 h and dissected under a microscope in 0.01 m PBS. Following dissection of individual quarter to half turns, all sections were stained with a 1:100 dilution of Rhodamine Phalloidin dye (Invitrogen R-415, Thermo Fisher Scientific) for 2 h, and then mounted on drop-slides in glycerol. Scout images were obtained at 50×, and custom software determined the distance from the apex and approximate frequency mapping. Chord lengths were measured at the location of the inner pillar cells on each scout image. Subsequent higher magnification images were then obtained at either 200× or 400× using either fluorescent microscopy or laser scanning microscopy. Hair cells were counted only when the stereocilia pattern could be visualized on the apical surface of the cell. Thirty hair-cell-wide bins (the approximate number visible in a single image at 400×) were counted and the percent missing from each bin were recorded at six separate cochlear regions per exposure group.

### Statistical analysis

Table 1 reports the number of mice that underwent ABR, DPOAE, and IC recordings, and the number of IC units included in each measure of neural sound processing. Mice with DPOAE recordings were a subset of mice with ABR recordings; however, mice that underwent IC recordings did not always have peripheral assessment. Auditory processing measures are reported as mean ± SEM, and statistical comparisons were made using GraphPad Prism. The Student’s *t* test compared differences between two groups, while ANOVA with Bonferroni *post hoc* testing compared the effect of one or more variables. χ^2^ tests were used to examine differences between observed and expected counts. Significance was set at *p *<* *0.05.

**Table 1:** Counts of animals tested and units isolated

	Control	Regular AAE	T-AAE
Periphery (# mice)			
ABRs	20	16	15
DPOAEs	13	12	8
			
Auditory midbrain			
# mice	11	11	10
Total # units recorded	825	932	904
FRAs (%)	574 (69.6)	702 (75.3)	629 (69.6)
80-dB gap responsive (%)	507 (61.5)	595 (63.8)	567 (62.7)
80-dB phasic units (%)	242 (29.3)	192 (20.6)	267 (29.5)
80-dB gap-in-noise responsive (%)	173 (21.0)	134 (14.4)	210 (23.2)
70-dB phasic units (%)	204 (24.7)	184 (19.7)	234 (25.9)
60-dB phasic units (%)	96 (11.6)	170 (18.2)	188 (20.8)

For peripheral measures, counts are given in terms of number of animals tested. For central auditory recording, counts are listed in terms of number of animals as well as the number units recorded from these animals. Percent values are listed as percent of total units recorded for each exposure type. All measure of dB listed are in dB SPL.

## Results

### Peripheral auditory assessment

**Figure 2.**
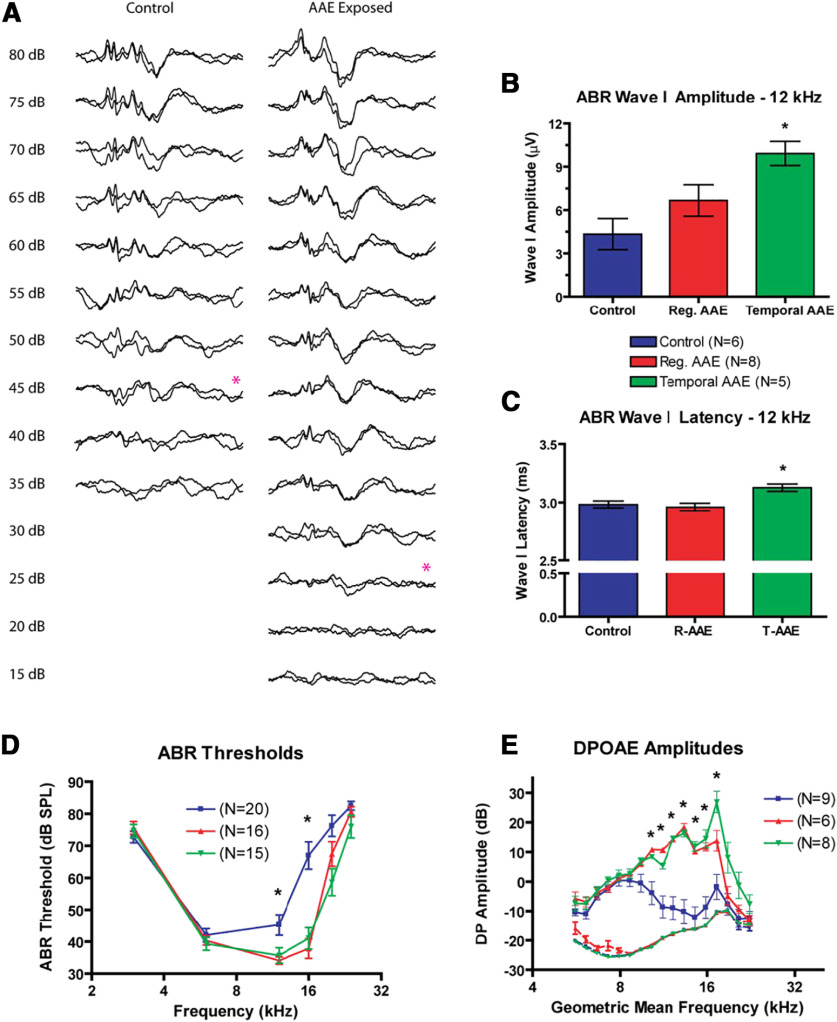
AAE exposure improves peripheral function at P30. ***A***, Representative ABR waveforms from a control and AAE-exposed animal at 12 kHz show similar suprathreshold morphology, with an elevated threshold in the control animal. Visual detection thresholds are denoted by the red asterisk. ***B***, ABR Wave I amplitudes were increased in both exposure types compared with controls [control (blue): 4.3 ± 1.1 μV; regular AAE (red): 6.7 ± 1.1 μV; T-AAE (green): 9.9 ± 0.8 μV], although only responses from T-AAE exposure reached significance (*p *<* *0.05). ***C***, ABR Wave I latencies were similar in magnitude (control: 2.98 ± 0.03 ms; regular AAE: 2.96 ± 0.03 ms; T-AAE: 3.13 ± 0.03 ms). T-AAE-exposed mice had significantly longer latency compared with control mice (*p* < 0.05) and regular AAE mice (*p *<* *0.01). ***D***, ABR thresholds were significantly decreased at 12 and 16 kHz following exposure to both types of AAE (regular = red, temporal = green) compared with controls (blue, *p *<* *0.001). At the frequency of greatest differences (16 kHz), this difference approaches 30 dB SPL. No responses were noted above 24 kHz in any group. ***E***, DPOAE amplitudes were increased following exposure to both types of AAE (regular = red, temporal = green) compared with controls (blue), with larger increases seen at select frequencies following T-AAE exposure. Between 10 and 17 kHz, both types of AAE exposure significantly increased amplitudes (mean increase: 21.2 ± 1.4 dB SPL, *p *<* *0.01). Additionally, T-AAE exposure resulted in a 13 dB SPL amplitude increase over regular AAE exposed mice at two test frequencies (17.27 and 18.84 kHz). Amplitudes could not be distinguished from the noise floor (shown in dashed lines, same colors as above) above 22 kHz in any group. In all group comparisons between treatment vs control groups significance denoted by * denotes *p *<* *0.05.

ABRs were recorded to determine the effect of early AAE exposure beginning at the onset of hearing. ABR thresholds were assessed as a function of Exposure and Frequency (Fig. 2*A*,*D*). A two-way ANOVA demonstrated significant effects of exposure (*F *=* *23.46, *p *<* *0.001), frequency (*F *=* *121.10, *p *<* *0.001), and exposure × frequency (*F *=* *5.71, *p *<* *0.001) with post-AAE thresholds from exposed mice being significantly improved as compared with control mice. *Post hoc* analysis showed significant improvement in ABR thresholds at 12 and 16 kHz following either type of AAE exposure, and no significant differences between the two types of AAE exposure. The magnitude of the difference in ABR thresholds for control and AAE-exposed mice approached 30 dB SPL at 16 kHz (control vs regular AAE: 29 dB SPL; control vs T-AAE: 26 dB SPL), a frequency in the range of the best hearing for DBA mice. While the difference did not reach statistical significance, at 24 kHz, we encountered very few mice from the control group that had observable responses at 80 dB SPL (3/20, or 15%), when compared with the regular AAE (6/16, or 38%) or T-AAE groups (7/15, or 46%). Together, these findings indicate that ABR thresholds improved following exposure to both types of AAE (Fig. 2*A*,*D*), replicating the findings of Turner and Willott (1998). Moreover, the frequency range that showed the most improvement in ABR thresholds was within the region of maximal energy for the AAE exposure spectrum and with the frequency region of best hearing sensitivity.

The amplitude and latency of wave 1 in the ABR waveform was measured in response to a 12-kHz tone presented at 80 dB SPL (Fig. 2*B*,*C*). Wave I amplitudes (Fig. 2*B*) demonstrated a significant effect of exposure (*F *=* *5.94, *p *=* *0.0118). *Post hoc* group comparisons reveal Wave I amplitudes were significantly greater for T-AAE-exposed mice than controls (9.9 ± 0.8 μV compared with 4.3 ± 1.1 μV, *p *<* *0.05). Mean Wave I amplitude was also greater for regular AAE-exposed mice (6.7 ± 1.1 μV) than controls, although the difference did not reach significance. Although the mean Wave I latencies were physiologically similar across groups, differing by only 170 μs (control: 2.98 ± 0.03 ms; regular AAE: 2.96 ± 0.03 ms; T-AAE: 3.13 ± 0.03 ms; Fig. 2*C*) the one-way ANOVA demonstrated a significant effect of exposure (*F *=* *6.81, *p *=* *0.007). Together these findings indicate the T-AAE exposure had a more substantive effect on Wave I ABR measures of cochlear sound processing than regular AAE exposure.

DPOAE amplitudes were measured in mice following exposure to regular AAE or T-AAE versus control environments to assess OHC function (Fig. 2*E*). A two-way ANOVA found significant effects for exposure (*F *=* *109.00, *p *<* *0.001), frequency (*F *=* *17.98, *p *<* *0.001), and exposure × frequency (*F *=* *5.25, *p *<* *0.001). *Post hoc* group comparisons revealed that DPOAE amplitudes increased at geometric mean frequencies between 10 and 17 kHz for both groups of AAE exposure relative to control (mean increase for pooled AAE-exposure responses: 21.2 ± 1.4 dB SPL, *p *<* *0.05). T-AAE exposure resulted in an even larger impact on DPOAE amplitudes than regular AAE exposure. *Post hoc* group comparisons revealed that DPOAEs elicited by 17.27- and 18.84-kHz tones were significantly larger (by 13 dB SPL) in T-AAE-exposed animals compared with regular AAE-exposed animals (*p *<* *0.05). Thus, similar to the ABR analysis, the DPOAE analysis showed the greatest impact of AAE exposure on cochlear function for stimuli in the frequency region with maximal AAE energy. Additionally, T-AAE exposure was moderately more effective at improving DPOAE measures of cochlear function.

**Figure 3.**
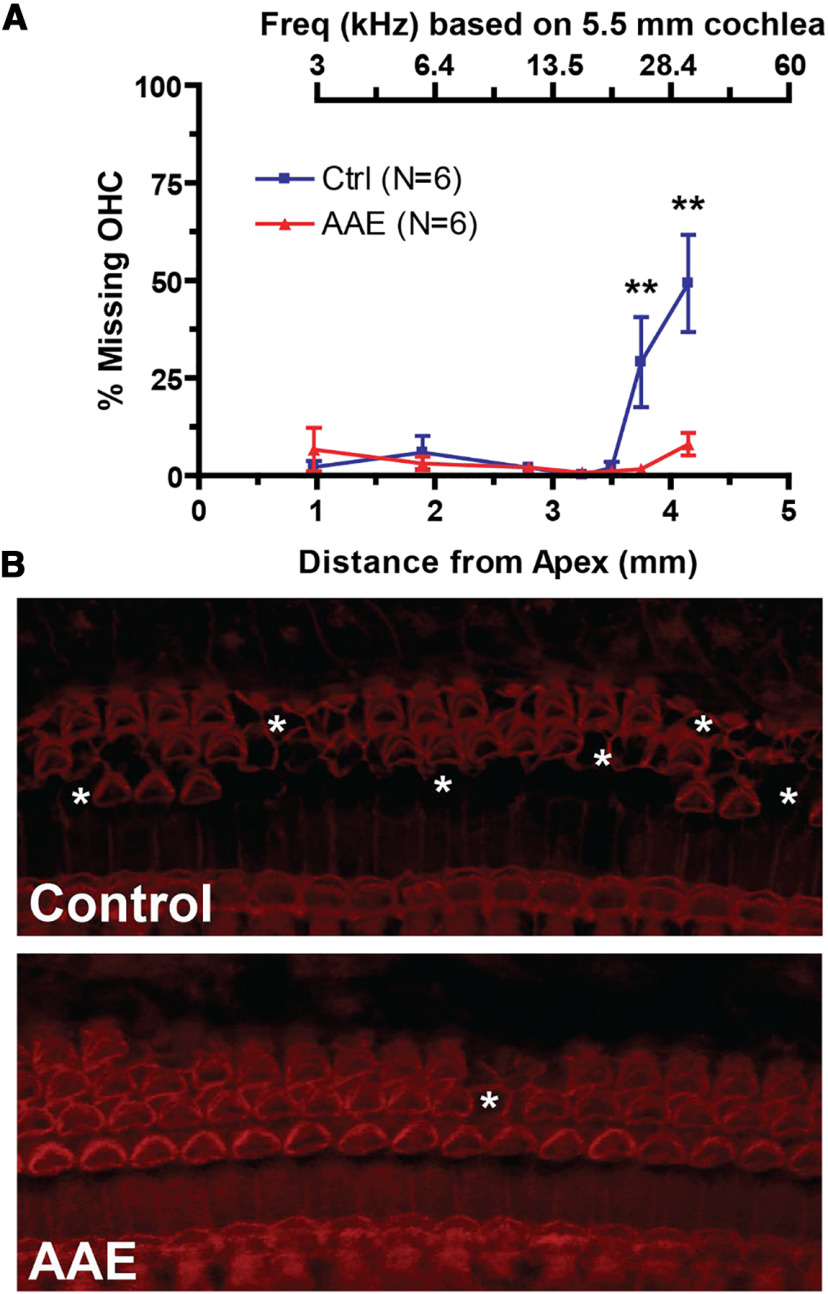
Mice exposed to AAE show significantly less OHC loss in the basal cochlea compared with control mice. ***A***, Percent missing OHCs versus the distance from apex (in mm) to the center of the counting bin. Approximate frequency mapping is shown above for a 5.5-mm mouse cochlea. Two-way ANOVA is significant for exposure, distance from apex, and interaction (*p *<* *0.001) while group comparisons show a significant increase in % missing OHCs at 3.75 mm from apex (29.1 ± 11.5% vs 1.7 ± 1.0%, *p *<* *0.01) and 4.15 mm from apex (49.3 ± 12.5% vs 8.1 ± 2.9%, **p <* *0.01). ***B***, Example composite laser scanning microscopy (LSM) images (400×) from control (top) and AAE-exposed (bottom) mice at the 3.75-mm region. Asterisks denote sections of missing OHCs. No inner hair cells were identified as missing for all animals in both AAE and control groups.

Previous work has shown that early AAE can spare hair cells in this mouse model of congenital hearing loss (Willott et al., 2005). Here, our DPOAE findings indicate that hair cell health is preserved in the present study as well. To confirm this, cochleae were examined in regular AAE-exposed and control mice. Regular AAE-exposed mice had significantly less OHC loss in the basal cochlea compared with control DBA mice (% loss at each distance: 3.75 mm: 1.7 ± 1.0% vs 29.1 ± 11.5%; 4.15 mm: 8.1 ± 2.9% vs 49.3 ± 12.5%, *p *<* *0.001; Fig. 3*A*). The mean basilar membrane length across all groups (*N* = 6 control DBA mice and 6 regular AAE-exposed mice) was 5.45 ± 0.1 mm. A representative frequency map is provided above the figure, based on a 5.5 mm cochlea using the Greenwood equation (Ou et al., 2000). Representative micrographs (Fig. 3*B*) show missing OHCs as those lacking stereocilia or cell body staining (Fig. 3*B*, asterisks). No inner hair cells were missing across all stained cochlear sections, from both exposed and control mice.

### Central auditory midbrain function

**Figure 4.**
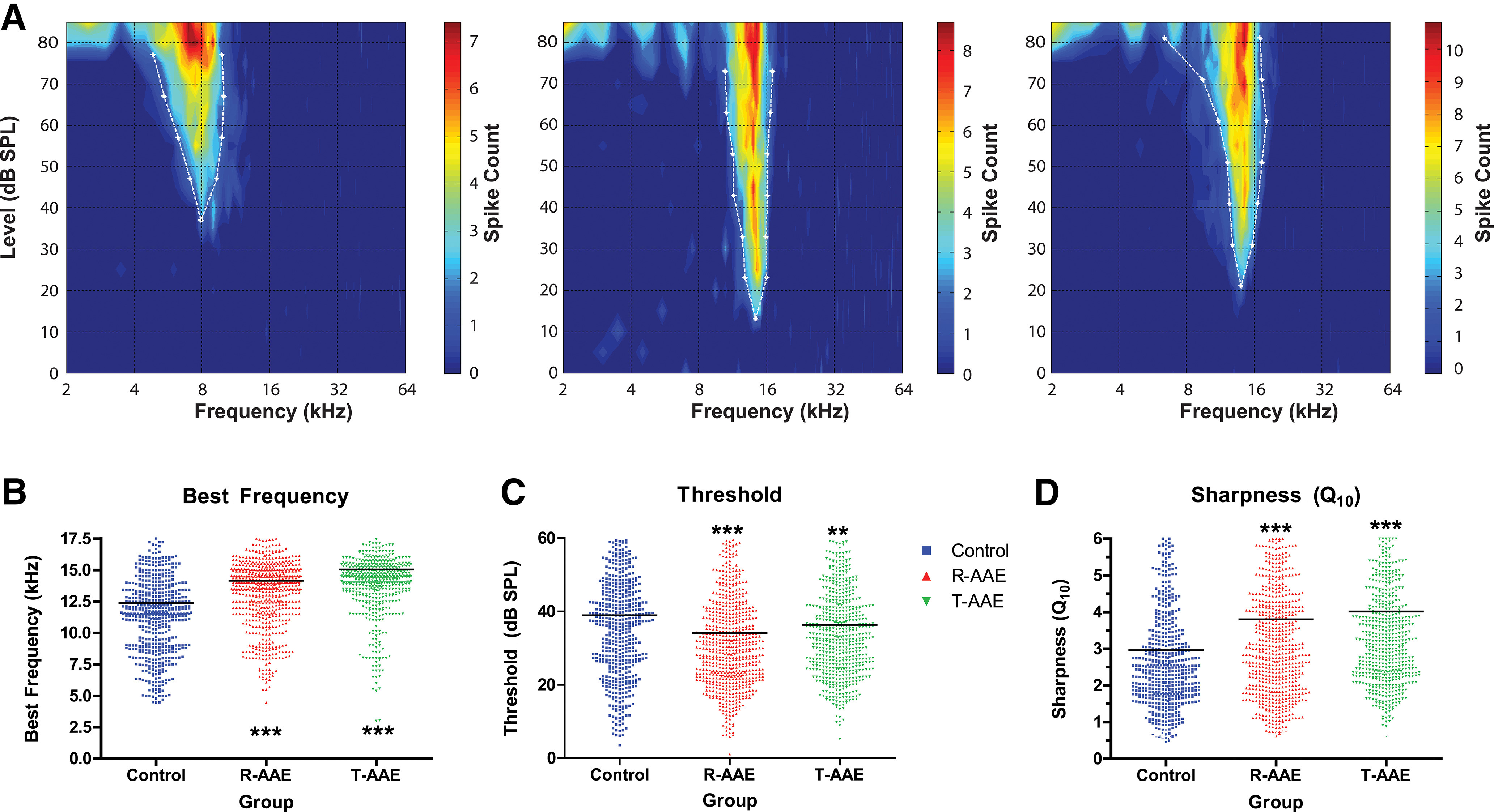
Exposure to AAE changes the frequency responses of IC units. ***A***, Example FRAs are shown from representative control, regular AAE-exposed, and T-AAE-exposed animals. Color-mapped counts indicate the number of spikes per frequency-level pair, with the legend shown on the right. BF was automatically identified as the frequency with the lowest intensity of drive (minimum threshold; MT). ***B***, Mean BFs were significantly increased compared with controls following either type of AAE exposure (regular AAE: 14.2 ± 0.2 kHz, T-AAE: 15.1 ± 0.2 kHz, control: 12.4 ± 0.2 kHz; *p *<* *0.001). Dot plot with a single point for each unit; black bar denotes mean value. No significant difference was seen between AAE exposure types. ***C***, Mean unit thresholds were significantly decreased following either type of AAE exposure (regular AAE: 34.2 ± 0.6 dB SPL, T-AAE: 35.9 ± 0.5 dB SPL, control: 38.9 ± 0.7 dB SPL; *p *<* *0.01). Again, no significant difference was seen between AAE exposure types. ***D***, Tuning sharpness was improved with both types of AAE exposure. Q values computed at 10 dB as well as 20, 30, and 40 dB above threshold (data not shown) were significantly increased compared with controls (one-way ANOVA with *post hoc* testing, *p *<* *0.001 at all levels). No significant differences were seen between the two types of AAE exposure. Graphs in ***B–D*** were vertically scaled to demonstrate differences in the mean, and thus some data points above the maximum vertical axis value are not shown and asterisks denotes significance between groups *p < 0.05, **p < 0.01, ***p < 0.001.

To determine whether AAE exposure can influence neural markers of spectral or temporal auditory processing acuity, we measured the response of IC neurons to sound stimuli in vivo. To our knowledge this is the first description of the effects of AAE on neural coding of complex sounds in the CAS. Exposure to AAE altered the frequency response properties for IC units when compared with units from control, unexposed animals (Fig. 4*A–D*). One-way ANOVAs demonstrated that exposure to AAE resulted in a significant upward shift in BF (*F *=* *43.16, *p *<* *0.001) and a significant improvement in minimum threshold (*F *=* *15.46, *p *<* *0.001) of neurons from AAE groups. Group comparisons revealed that both types of AAE exposure significantly increased the upward frequency boundary of BFs relative to control values [regular AAE (*N* = 702): 14.2 ± 0.2 kHz; T-AAE (*N* = 629): 15.1 ± 0.2 kHz; control (*N* = 574): 12.4 ± 0.2 kHz; *p *<* *0.001], with no further differences between regular AAE and T-AAE exposure groups (Fig. 4*B*). Likewise, both types of AAE exposure significantly improved minimal response threshold, with no further effect of AAE exposure group (Fig. 4*C*). Finally, exposure to either type of AAE sharpened tuning of the FRAs, assessed by measuring Q values for the FRA between 10 and 40 dB SPL above threshold. One-way ANOVAs demonstrated a significant effect of exposure for Q_10_ through Q_40_ values (Q_10_: *F *=* *28.81; Q_20_: *F *=* *43.46; Q_30_: *F *=* *34.09; Q_40_: *F *=* *48.58; *p *<* *0.001). A higher Q value indicates sharper tuning, and group comparisons showed that Q_10_ through Q_40_ values following either type of AAE exposure were higher than control values (Q_10_ shown in Fig. 4*D*). The mean magnitude of the difference between control and AAE exposure groups at Q_10_ is ∼1, which at a BF of 12 kHz equates to a bandwidth difference of ∼1 kHz (or 25% of the control group mean). No other significant differences were found with respect to frequency receptive fields between exposure types. These findings indicate that in AAE-exposed mice IC neurons had lower thresholds and were more responsive to higher frequencies, but were also more narrowly tuned. The type of AAE exposure did not influence these improvements in spectral acuity.

**Figure 5.**
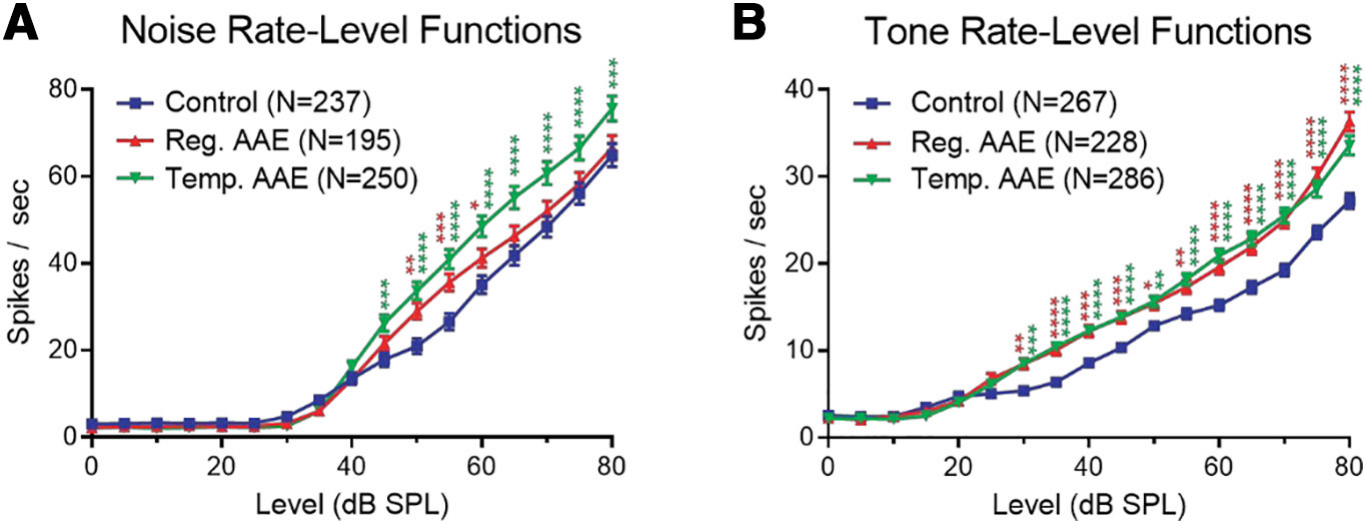
AAE exposure increases excitatory drive in the auditory midbrain as measured by rate-level functions. ***A***, IC unit responses (spikes per second) to noise bursts (25 ms) plotted as a function of intensity (dB SPL) showed that AAE exposure significantly enhanced responses to moderate and/or high intensity wide-band noise. Two-way RM ANOVA showed a significant effect of intensity, as expected (*F*_(16,11543)_ = 591.3; *p *<* *0.0001), as well as a significant effect of treatment group (*F*_(2,11543)_ = 45.85; *p *<* *0.0001). *Post hoc* comparisons with Bonferroni correction showed significantly enhanced responses at intensities ≥45 dB SPL following regular (red asterisks) and temporal (green asterisks) AAE relative to controls (*p < 0.05, **p < 0.01, ***p < 0.001) . The T-AAE group also had significantly larger responses than the regular AAE group to stimuli ≥60 dB SPL (not demarcated for clarity). ***B***, AAE exposure also enhanced IC unit responses to moderate and high intensities tone bursts. Tone bursts (25 ms) were delivered at the unit’s BF, identified as the frequency with the lowest intensity of drive. Responses to BF tone bursts (spikes per second) were plotted as a function of intensity (dB SPL). As expected, there was significant effect of intensity (*F*_(16,13226)_ = 699.5; *p *<* *0.0001), and there was also a significant effect of treatment group (*F*_(2,13226)_ = 129.2; *p *<* *0.0001). *Post hoc* comparisons showed significantly enhanced responses at intensities ≥30 dB SPL following regular (red asterisks) and temporal (green asterisks) AAE relative to controls. The regular AAE group also had significantly larger responses to 80 dB SPL stimuli than the T-AAE group (not demarcated for clarity).

Rate-intensity coding was assessed in response to both noise and tone stimuli. Temporal and regular AAE exposure enhanced growth in population rate-intensity functions relative to the unexposed control group (Fig. 5*A*,*B*). For wide-band noise stimuli, regular AAE enhanced driven responses at moderate intensities (45–60 dB SPL) relative to controls, while T-AAE increased responses at both these moderate and high intensities (Fig. 5*A*). In contrast, when tone stimuli were presented at BF, a similar enhancement in rate-intensity function growth was observed following both regular AAE and T-AAE relative to controls (Fig. 5*B*). These findings continue to support the conclusion that the type of AAE exposure did not strongly influence the improvement in spectral acuity. However, driven rates were greater for noise stimuli, which were spectrally similar to the AAE stimulus, than for tone stimuli.

**Figure 6.**
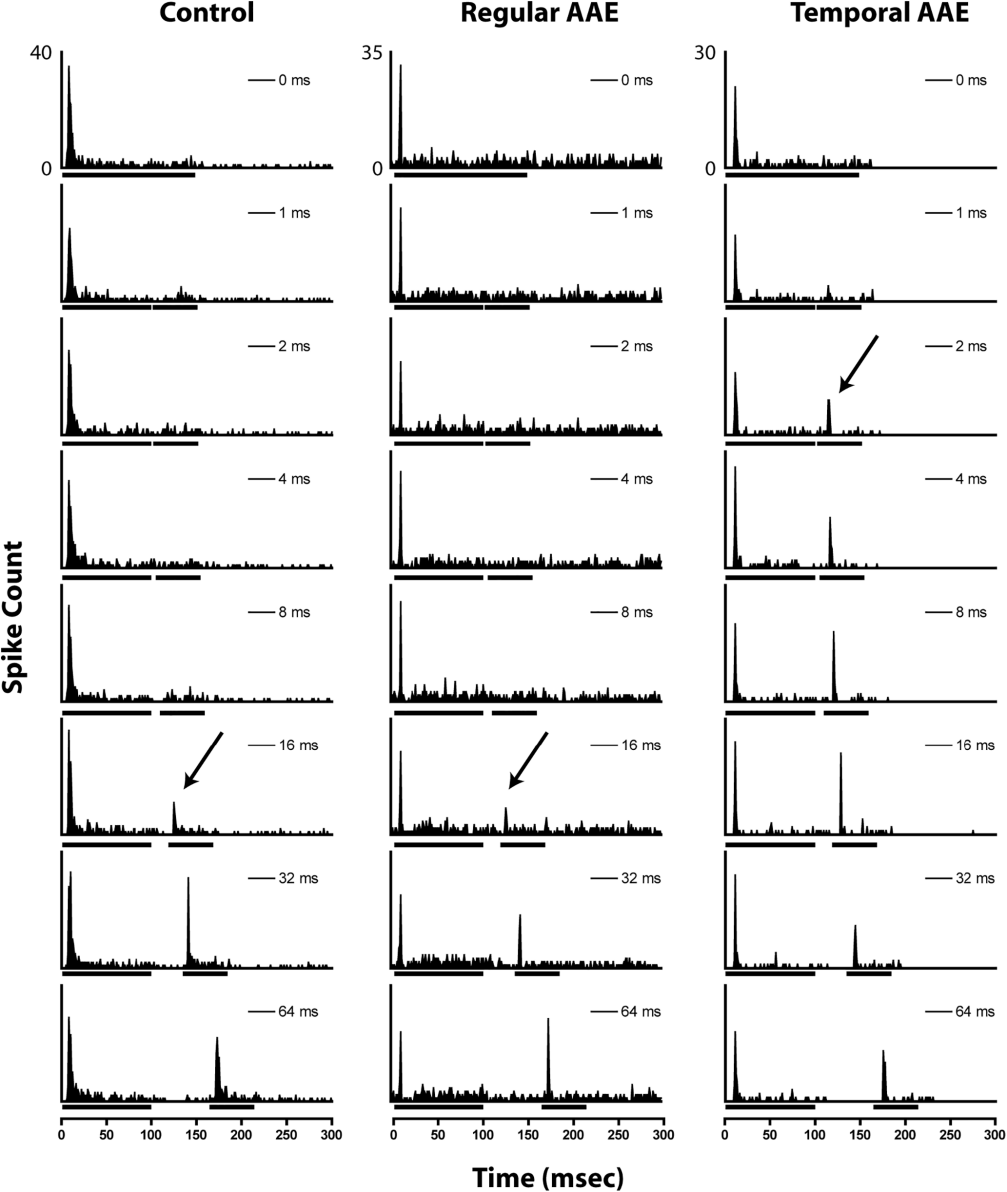
Representative examples of gap encoding of phasic units from unexposed (left), regular AAE-exposed (center), and T-AAE-exposed (right) mice. Bars under the *x*-axis denote noise-burst duration marking the silent gap. Arrows denote the automatically-calculated MGT for each unit.

**Figure 7.**
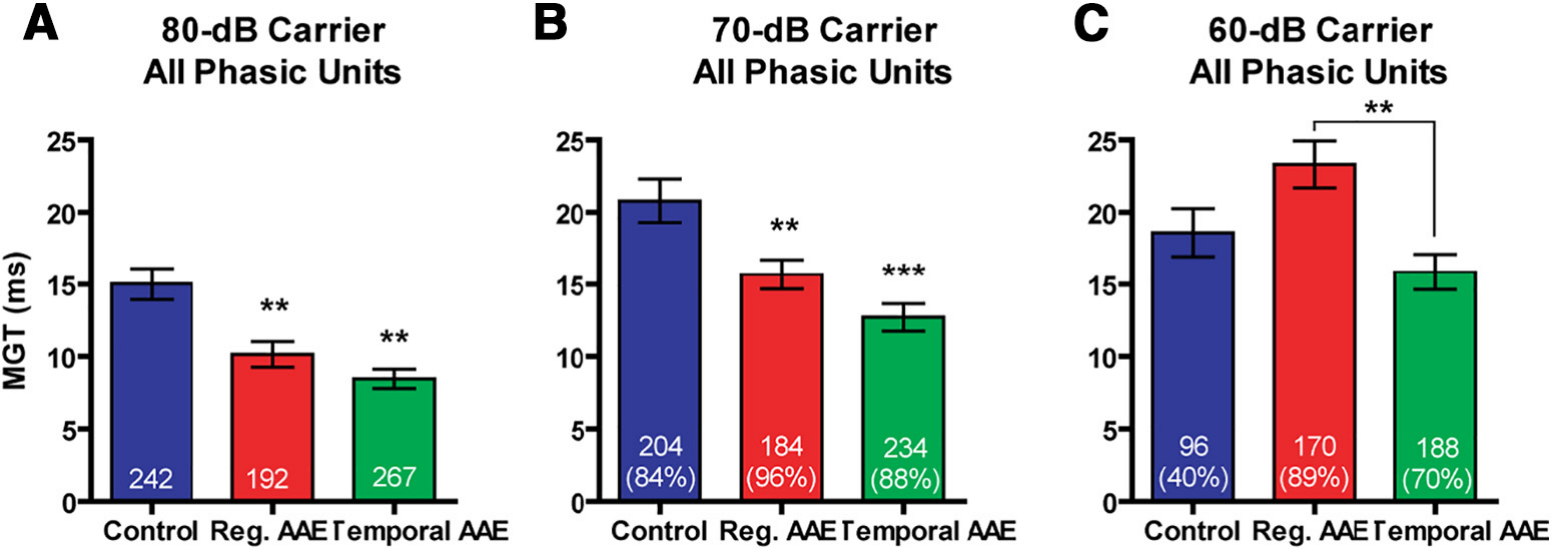
Exposure to both types of AAE improve mean gap thresholds (MGTs) in phasic units. MGTs were computed across each group, for each noise carrier level (80, 70, and 60 dB SPL). ***A***, At the 80 dB SPL carrier level exposure to both types of AAE resulted in shorter mean MGT (control: 15.0 ± 1.1 ms, regular AAE: 10.1 ± 0.9, T-AAE: 8.4 ± 0.7 ms). ***B***, At the 70 dB SPL carrier level, exposure to both types of AAE again shorten mean MGT, with greater improvement seen in the T-AAE exposure group (control: 20.8 ± 1.5 ms, regular AAE: 15.7 ± 1.0, T-AAE: 12.7 ± 1.0 ms). ***C***, At the 60 dB SPL carrier level, the mean MGT from the T-AAE group was significantly shorter than from the regular AAE group (15.9 ± 1.2 vs 23.3 ± 1.6 ms, *p *<* *0.01). Additionally, the mean MGT for control mice was significantly shorter than from the regular AAE group (18.6 ± 1.7 vs 23.3 ± 1.6 ms), but the number of responsive units was much less. Sample size is shown inside the bar, with the percent shown as a percent of all phasic responsive units to an 80 dB SPL carrier. Asterisks denote a significant difference between regular or T-AAE and controls, **p < 0.01, ***p < 0.001.

Temporal processing acuity was assessed via MGTs, a measure of neural coding of silent gaps embedded in noise. Representative poststimulus time histograms (PSTHs) of single phasic units in response to different gap durations are shown in Figure 6. MGTs were computed for all phasic units, and the unit was included in each of the following analyses if the gap threshold for the condition was ≤96 ms [*N* (% of all units recorded), control: 242 (42%); regular AAE: 192 (27%); T-AAE: 267 (42%)]. For gaps embedded in 80 dB SPL noise carriers, one-way ANOVA demonstrated a significant effect of exposure (*F *=* *15.43, *p *<* *0.001; Fig. 7*A*). Both types of AAE exposure shortened MGTs relative to controls. The mean magnitude of improvement for the regular AAE exposure group was 4.89 ms (33%), and for the T-AAE exposure group was 6.58 ms (44%). For 70 dB SPL carriers, one-way ANOVA demonstrated a significant effect of exposure (*F *=* *12.49, *p *<* *0.001). Again, both types of AAE exposure improved MGTs compared with controls, with greater average improvement seen by mice exposed to T-AAE (8.10 ms) versus those exposed to regular AAE (5.11 ms). *Post hoc* comparison was not significantly different between groups exposed to regular AAE versus T-AAE. For silent gaps embedded in 60 dB SPL carriers, one-way ANOVA demonstrated an effect of exposure on MGT (*F *=* *7.31, *p *<* *0.001). Group comparisons show that mice exposed to T-AAE had significantly shorter MGTs compared with mice exposed to regular AAE (T-AAE vs regular AAE: 15.9 ± 1.2 vs 23.3 ± 1.6 ms, *p* < 0.01). Control mice also had shorter MGTs compared with mice exposed to regular AAE however these differences did not reach significance (control vs regular AAE: 18.6 ± 1.7 vs 23.3 ± 1.6 ms). However, the number of units from control mice with detectable minimal gap thresholds (96 units) was also substantially lower than units from both regular (170 units) and temporal (188 units) AAE-exposed mice (χ^2^(2) = 118.08, *p *<* *0.001). In contrast with phasic units, tonic units showed no significant effects of AAE exposure on responses to gap stimuli or MGTs (data not shown). Overall, these findings indicate that gap detection generally improved in phasic units following exposure to both types of AAE, with a trend toward greater improvement seen following exposure to our novel T-AAE.

**Figure 8.**
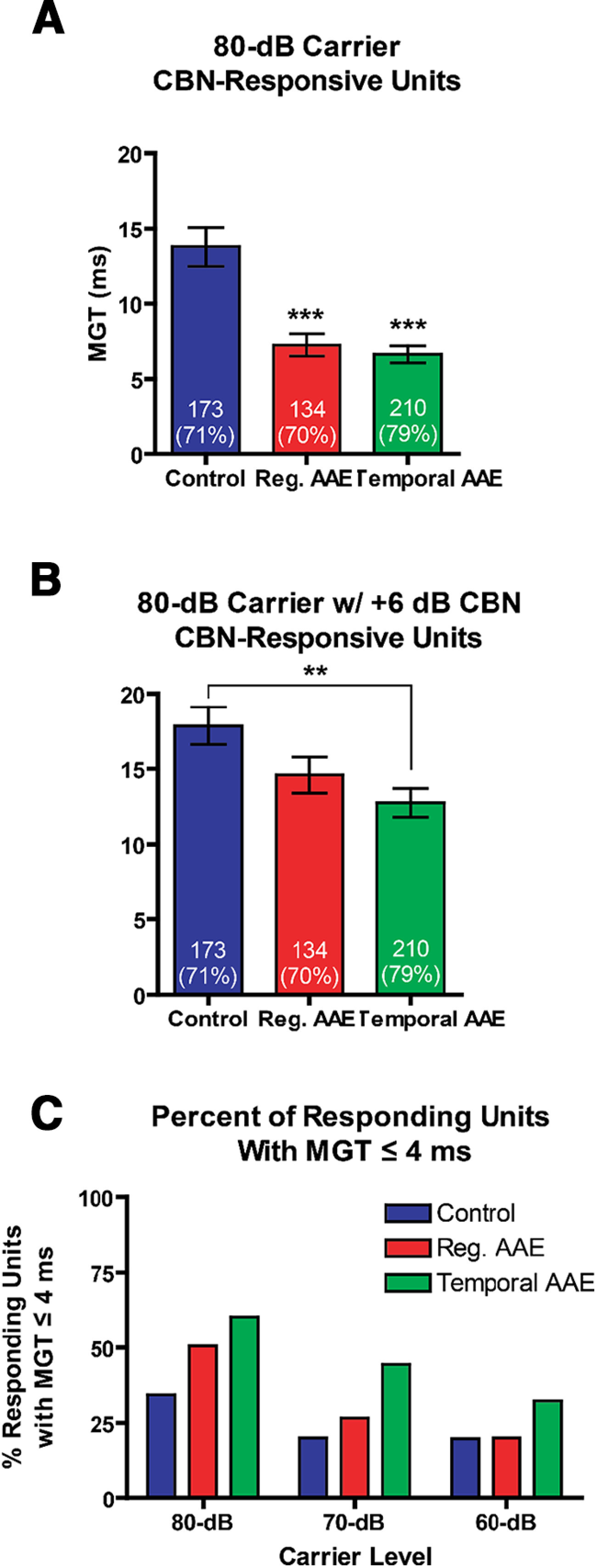
Exposure to T-AAE preserves gap thresholds in the presence of CBN. Only a subset of phasic units was responsive in background noise. ***A***, Shown are the MGTs measured in response to an 80 dB SPL carrier without CBN from only units responsive in background noise. These results match those seen in Figure 5*A*, where at 80 dB SPL, both types of AAE exposure resulted in significantly shorter MGT (***p < .001). ***B***, The same units as in ***A***, measured in response to an 80 dB SPL carrier with +6 dB SNR CBN (background noise at 74 dB). Only exposure to T-AAE resulted in significantly shorter gap thresholds compared with controls (12.7 ± 1.0 vs 17.9 ± 1.2 ms, **p < 0.01). Sample size is given in each bar, with percent shown as a percent of all phasic responders at 80 dB SPL. ***C***, The percent of all responsive phasic units with MGTs ≤4 ms (sensitive responders) was increased in the T-AAE group at all levels. Asterisks denote a significant difference between regular or T-AAE and controls, **p <* *0.01; ***p <* *0.001.

Gap detection is more challenging in background noise, and may also be a key marker of speech recognition difficulties in background noise (Snell and Frisina, 2000; Pichora-Fuller et al., 2006). To determine whether this measure of temporal acuity improves following AAE exposure, only units that responded to gap stimuli (MGTs ≤ 96 ms) presented in CBN were included in the analysis (Table 1; Fig. 8*A*,*B*). This subpopulation also showed improvement in MGTs for gap stimuli presented in quiet after either AAE exposure (one-way ANOVA: *F *=* *18.16, *p *<* *0.001; *post hoc* regular AAE and T-AAE MGT < control, *p *<* *0.001; compare Figs. 8*A* and 6*A*). A one-way ANOVA also showed a significant effect of Exposure on MGTs when stimuli were delivered in the presence of +6 dB SNR CBN (*F *=* *5.39, *p *=* *0.005; Fig. 8*B*). Exposure to T-AAE significantly shortened MGTs compared with controls (12.7 ± 1.0 vs 17.9 ± 1.2 ms, *p *<* *0.01), while exposure to regular AAE only trended toward shorter MGTs (14.6 ± 1.2 vs 17.9 ± 1.2 ms, *p *>* *0.05). These data indicate that early T-AAE exposure improves gap detection in the presence of background noise for phasic units.

As our previous work suggests that behavioral gap thresholds are more strongly influenced by midbrain units with the shortest gap thresholds (Barsz et al., 2002), we computed the percent of responding units with gap thresholds ≤4 ms (Fig. 8*C*). All phasic units with MGT ≤ 96 ms (those shown in Fig. 7*A–C*) were included for this analysis. T-AAE-exposed mice had the greatest percent of phasic units that had short gap thresholds, regardless of carrier intensity. Thus, early T-AAE strengthens encoding of short gap durations.

## Discussion

In the present study, we have shown that exposure to a temporally-complex broadband AAE can modulate multiple aspects of peripheral and central auditory function in a mouse model of severe congenital SNHL. Early exposure to a novel, temporally-enriched broadband noise stimulus, starting before hearing onset, improved ABR thresholds, Wave I ABR amplitudes and DPOAE amplitudes relative to normally-raised mice. The frequency range that showed the most improvement in cochlear function was within the region of maximal energy for the AAE exposure spectrum. Improvements in sensitivity and spectral encoding were also present in the CAS. Recordings in the auditory midbrain showed lower neural thresholds to sounds and better representation of higher frequencies following an enriched versus control environment. Importantly, neural gap detection improved in both quiet and background noise, indicating increased temporal processing acuity after this novel AAE intervention. Together, these findings suggest that early exposure to temporally-modulated broadband noise stimuli can restrict the negative consequences of SNHL on peripheral function, and spectral and static temporal processing in the CAS.

Sensorineural damage in the cochlea decreases sensitivity and distorts auditory input from the periphery (Schmiedt, 2010). Consequent to this peripheral damage, a number of structural and neurophysiological changes occur in brainstem, midbrain, and cortical auditory brain regions (Casey, 1990; Caspary et al., 1990, 2008; Gleich et al., 1994; Kilgard and Merzenich, 1998; Krenning et al., 1998; Helfert et al., 1999, 2003; Wang and Manis, 2005; Frisina and Walton, 2006). Altered central auditory processing associated with SNHL, such high thresholds, loss of frequency representation, and broader tuning curves (Turner and Willott, 1998; Barsz et al., 2007), may impair auditory signal detection and differentiation. Additionally, very early SNHL, experienced in DBA mice and infants with congenital SNHL, may interact with normal developmental timelines for central auditory processing to further impair sound processing beyond the direct consequences of SNHL. In normal hearing humans and rodents, temporal processing acuity, as measured by gap thresholds, improves during early development (Trehub et al., 1995; Friedman et al., 2004). Here, we find deficits in neural correlates of gap detection in the auditory midbrain in one-month-old DBA mice relative to normal hearing strains, such as the CBA/CaJ (Barsz et al., 2002; Walton et al., 2008). Thus, while SNHL in adulthood does not have profound effects on temporal processing in the auditory midbrain (Walton et al., 2008), the current findings suggest that SNHL during development may strongly impact both spectral and temporal aspects of central auditory processing. Interestingly, Burghard et al. (2019) found that Cdh23 mutant mice, a mouse strain that shows early hearing loss, also showed declines in neural temporal coding of amplitude modulation. However, the decline in AM processing was independent of threshold loss given that heterozygotes exhibited the former without the latter. Although the study did not address gap encoding, as is addressed here, the findings suggest that reducing hearing threshold loss alone may not be sufficient to save temporal processing.

Previous work indicates that in young mice with progressive, congenital SNHL, early exposure to broadband AAE can preserve hearing sensitivity and limit hair cell loss (Turner and Willott, 1998; Willott and Turner, 1999, 2000; Willott et al., 2005, 2006). This also appears to limit concomitant reorganization in the CAS, limiting the loss of neurons in the cochlear nucleus and sensitivity to high-frequency sounds in the IC (Willott and Turner, 2000; Willott et al., 2005). Here, we expand this characterization of central auditory processing after early temporally modulated broadband AAE exposure, finding that intervention can preserve both spectral and temporal acuity in the auditory midbrain. After AAE intervention with our novel temporally complex AAE, and to a lesser extent with a less complex broadband AAE, FRAs of IC neurons exhibited improved minimal response thresholds and improved high-frequency encoding, sharper FRAs, and improved gap encoding. In addition, increased excitatory drive as measured by rate-intensity functions was observed. Others have reported similar CAS plasticity following more general environmental enrichment with an auditory component. Recordings in the auditory cortex (AC) showed improved neural temporal response properties, increased spectral and temporal selectivity, and more narrow neural response fields (Cai et al., 2009; Jakkamsetti et al., 2012). The current findings continue to support the idea that both peripheral and central auditory processing can be modulated by enriched environments.

The plasticity of the CAS is remarkable across mammalian species. Like rodents, humans exposed to various types of passive AAE that alter sound input to the ear also undergo profound central auditory and perceptual changes resulting from neural plasticity. The effects of AAE on central auditory function, particularly in the face of hearing loss, may arise, at least in part, from homeostatic mechanisms that maintain neural activity (Turrigiano and Nelson, 2004). Consistent with this idea, when sound input is attenuated via deprivation (i.e., via temporary earplug, or conductive hearing loss), the reduced peripheral input leads to increased central activity (see Noreña and Farley, 2013). Subsequent (or concurrent) exposure to passive AAE is predicted to stabilize peripheral excitatory drive and preserve input to the CAS. By stabilizing the mean level of neural activity, AAE is predicted to counteract hearing loss-related increases in central gain, improving coding efficiency, and maintaining an optimal input-dependent dynamic range (Eggermont, 2013). Indeed, perceptual changes in humans are observed following AAE that are consistent with normalized gain, including altered loudness perception (Formby et al., 2003; Munro et al., 2014), and finer intensity resolution (Philibert et al., 2002). AAE may also help to maintain or expand sound representation in the face of deteriorating peripheral input through standard experience-dependent plasticity mechanisms. Humans show improved temporal coding following AAE (Gabriel et al., 2006), which could arise from improved sound representation. In rodents, AAE can lead to reorganization in primary and non-primary auditory cortex as reflected in narrower response fields, improved temporal response properties, and increased spectral and temporal selectivity of neurons (Cai et al., 2010; Jakkamsetti et al., 2012).

Intriguingly, while both types of passive AAE employed in this study improved auditory sensitivity and spectral sound processing, our novel, temporally complex broadband AAE had a stronger positive influence on temporal processing acuity, both in quiet and in background noise. This was particularly true for improvements in temporal processing with background noise. Thus, the benefits of early AAE exposure may be related to characteristics of the sound presented. Previous work showed that in young DBA mice, treatment with broadband AAE improved behavioral and neurophysiological measures of tonal thresholds (Turner and Willott, 1998). In the present study, a similar, but temporally more complex broadband-AAE stimulus, improved both spectral and temporal sound processing. Band-limited AAE also slowed the progression of SNHL in the 16- to 32-kHz range, but did not ameliorate a loss of sensitivity at lower frequencies (Willott et al., 2006). Likewise, the effect of AAE exposure is also shaped by auditory function and timing of the intervention. In mature auditory systems, or with normal hearing, some types of AAE exposure may instead lead to the suppression of sound sensitivity (Willott and Turner, 2000; de Villers-Sidani et al., 2007). Recently it was observed that young adult CBA mice exposed to 75 dB SPL AAE were found to display functional evidence of cochlear synaptopathy (Pienkowski, 2019). Similarly, in adult cats with normal hearing, tonal or band-limited AAE exposure profoundly suppressed AC activity in the frequency range of the exposure (Pienkowski and Eggermont, 2009, 2010a,b; Pienkowski et al., 2011).

Although the precise mechanism is unknown, early broadband AAE has a positive impact on cochlear health across a limited tonotopic range depending on the spectral composition of the AAE (Willott et al., 2005). Improved cochlear function in turn supports lower thresholds and maintains frequency representation in the CAS. However, this study re-affirms that broadband AAE exposure invokes further adaptive neural plasticity in the auditory midbrain. We observed sharper IC neural tuning curves, which may result from enhanced lateral inhibition in the CAS following auditory enrichment. Moreover, although we did not test the role of inhibition in this study, we hypothesize that temporally complex AAE may not only strengthen inhibition, but also improve the timing of inhibition. Timed inhibition is key for shaping sound offset responses that subserve gap detection (Walton, 2010; Weible et al., 2014; Anderson and Linden, 2016; Kopp-Scheinpflug et al., 2018). Since more spectrally complex signals evoke stronger sound offset responses, the broadband nature of the stimulus might be important, and may even be improved using spectral components (Akimov et al., 2017). The possibility that temporal encoding can be altered by auditory experience was first confirmed by Kilgard and Merzenich (1998), who demonstrated that the ability of AC neurons to follow high-frequency sound stimuli can be improved if high-frequency sound stimuli are paired with electrical stimulation of the nucleus basalis. The improvement in neural encoding and detection of gaps in noise in the current study shows that even passive sound exposure may shape temporal acuity, although it seems unlikely that this occurs through pathways involving the nucleus basalis.

Overall, the current findings suggest that temporally-complex AAE interventions may provide functional benefits in individuals with SNHL, especially newborns diagnosed with hearing loss. The improved neural encoding of short gap durations in the IC is likely to support functional improvements in gap detection, as these measures are strongly associated (Barsz et al., 2002). In turn, improved gap detection, particularly in noise, counteracts an aspect of hidden hearing loss that impairs speech perception in daily life (Snell et al., 2002). Although not specific to the temporally complex AAE intervention, better frequency representation and sharper spectral tuning that occurs after AAE may also bolster auditory signal detection and differentiation. These findings support the possibility that AAE may be targeted, based on the properties of the AAE as well as the listener, to better improve hearing deficits. The ability of our novel, temporally complex, broadband AAE exposure to improve neural correlates of SNHL provides direct bench-to-bedside promise for treating congenital SNHL.

## Synthesis

Reviewing Editor: Christine Portfors, Washington State University Decisions are customarily a result of the Reviewing Editor and the peer reviewers coming together and discussing their recommendations until a consensus is reached. When revisions are invited, a fact-based synthesis statement explaining their decision and outlining what is needed to prepare a revision will be listed below. The following reviewer(s) agreed to reveal their identity: NONE. Note: If this manuscript was transferred from JNeurosci and a decision was made to accept the manuscript without peer review, a brief statement to this effect will instead be what is listed below.

Thank you for responding so thoroughly to the previous reviewers' comments.
